# Loss of the ferripyochelin receptor FptA drives reduced cefiderocol susceptibility and impairs fitness in *Pseudomonas aeruginosa* PA14

**DOI:** 10.1128/aac.01410-25

**Published:** 2026-02-12

**Authors:** Donghoon Kang, Rodrigo P. Baptista, Salvador I. Drusin, Diego M. Moreno, Cesar A. Arias, William R. Miller

**Affiliations:** 1 Division of Infectious Diseases, Houston Methodist Hospital 23534 , Houston, Texas, USA; 2 Center for Infectious Diseases, Houston Methodist Research Institute 167626 , Houston, Texas, USA; 3 Facultad de Ciencias Bioquímicas y Farmacéuticas, Universidad Nacional de Rosario 28237, Rosario, Santa Fe, Argentina; 4 Instituto de Química Rosario (IQUIR, CONICET-UNR), Rosario, Santa Fe, Argentina; 5 Department of Medicine, Weill Cornell Medical College 12295 , New York, New York, USA; Columbia University Irving Medical Center, New York, New York, USA

**Keywords:** *Pseudomonas aeruginosa*, antimicrobial resistance, cefiderocol, siderophores, pyoverdine, pyochelin

## Abstract

*Pseudomonas aeruginosa* is an opportunistic human pathogen and a frequent cause of multidrug-resistant infections. This organism continues to evade antimicrobial therapy despite the clinical introduction of new antipseudomonal antibiotics over the past several years. One of these agents is cefiderocol (FDC), a novel siderophore-cephalosporin conjugate antibiotic that was designed to overcome both intrinsic and acquired β-lactam resistance mechanisms in *P. aeruginosa*. However, studies have demonstrated that inactivation of energy transducer protein (TonB)-dependent receptors, most notably the catechol siderophore receptor *piuA,* can substantially curtail the drug’s ability to permeate the bacterial outer membrane, leading to rapid development of resistance. In this study, we examined the FDC resistance mechanisms of the laboratory strain PA14. We demonstrated that inactivation of the ferripyochelin receptor FptA was a first-step mutation toward FDC resistance. Through transposon mutagenesis, we identified several resistance pathways following *fptA* inactivation, such as the loss of an additional FDC receptor and overexpression of the MuxABC-OpmB multidrug efflux system. Introduction of clinically identified mutations analogous to these transposon insertions in the absence of *fptA* conferred full FDC non-susceptibility while preserving the activity of other antipseudomonal β-lactam antibiotics. We also demonstrated that inactivation of *fptA* in a pyoverdine biosynthetic mutant disrupted bacterial iron homeostasis and conferred a fitness disadvantage. These FDC resistance mechanisms identified in PA14 highlight the long-term challenges of using FDC treatment for drug-resistant *P. aeruginosa* infections.

## INTRODUCTION

*Pseudomonas aeruginosa* is a leading cause of healthcare-associated infections, particularly in critically ill individuals and those with chronic lung diseases such as cystic fibrosis (1). Therapy for infections due to these organisms is complicated by clinical isolates displaying difficult-to-treat resistance (DTR) that includes resistance to fluoroquinolones, piperacillin-tazobactam, cefepime, ceftazidime, aztreonam, and carbapenems (2). Rates of resistance to the newer β-lactam/β-lactamase inhibitor (BL/BLI) combinations such as ceftolozane-tazobactam, ceftazidime-avibactam, and imipenem-relebactam have also increased among global collections of extensively drug-resistant isolates (3, 4). Cefiderocol (FDC) is a siderophore-conjugated cephalosporin that retains *in vitro* activity against drug-resistant *P. aeruginosa* by utilizing TonB-dependent receptor iron transporters to facilitate drug uptake (5). Despite high rates of susceptibility *in vitro*, clinical failure and the emergence of resistance to FDC on therapy are growing concerns (6–8).

A number of mutations have been linked to FDC resistance from both clinical isolates before and after antibiotic exposure, as well as *in vitro* adaptation assays. These can be grouped into several categories. First, alterations of outer membrane TonB-dependent siderophore transporters or their regulatory components are postulated to act through inactivation or down-regulation of FDC sites of entry (9, 10). These changes may arise during FDC exposure, although decreased expression of the major catechol transporters *piuA* and *pirA* due to frameshift mutations in the transcriptional regulator *pirR* has been noted in the *P. aeruginosa* population prior to the introduction of FDC (11). PiuA and PirA are the primary receptors that allow *P. aeruginosa* to utilize plant-derived catechol molecules under iron-restrictive conditions (10) and have been characterized as the main TonB-dependent receptors that facilitate entry of several siderophore-conjugated drugs, including FDC, in the laboratory strain PAO1 (12–14). The two-component sensor regulator PirR promotes *piuA* and *pirA* expressions upon induction by catechol compounds (10). Second, mutations leading to increased activation of the CpxS histidine kinase have been demonstrated to increase the expression of two efflux systems, MexAB-OprM and MuxABC-OpmB (15, 16), possibly resulting in antibiotic efflux or increased secretion of pyoverdine (17). Finally, the presence of certain acquired β-lactamases or mutations in the intrinsic pseudomonal AmpC cephalosporinase has been associated with FDC resistance (18, 19) and may be selected by prior exposure to ceftolozane-tazobactam or ceftazidime-avibactam (20, 21).

*P. aeruginosa* possesses two endogenous siderophores with different affinities for ferric iron, pyoverdine and pyochelin. Pyoverdine has a distinctly high affinity for ferric iron (K_d_ = 10^−32^ M) (22), and *in vitro* data suggest that it may chelate the metal from FDC (5), preventing the antibiotic from utilizing TonB-dependent receptors for cell entry (17). Conversely, pyochelin has a lower iron affinity, and production of this siderophore leads to upregulation of the cognate TonB-receptor FptA (23, 24). Overexpression of FptA results in increased susceptibility to FDC, likely through increased antibiotic uptake (9), and we have previously identified *fptA*-inactivating mutations in laboratory and clinical isolates that developed FDC resistance during therapy (6, 11). Notably, we recently observed treatment-emergent FDC nonsusceptibility in *P. aeruginosa,* mediated by *fptA* inactivation without additional mutations targeting the catechol transporters PiuA or PirA (6). However, the specific contribution of *fptA* mutations to the FDC resistance phenotype has not been addressed. The aim of this study was to characterize the acquisition and impact of *fptA* mutations on the FDC susceptibility and fitness of the laboratory strain *P. aeruginosa* PA14.

## RESULTS

### FptA is a presumptive receptor for FDC

**Fig 1:**
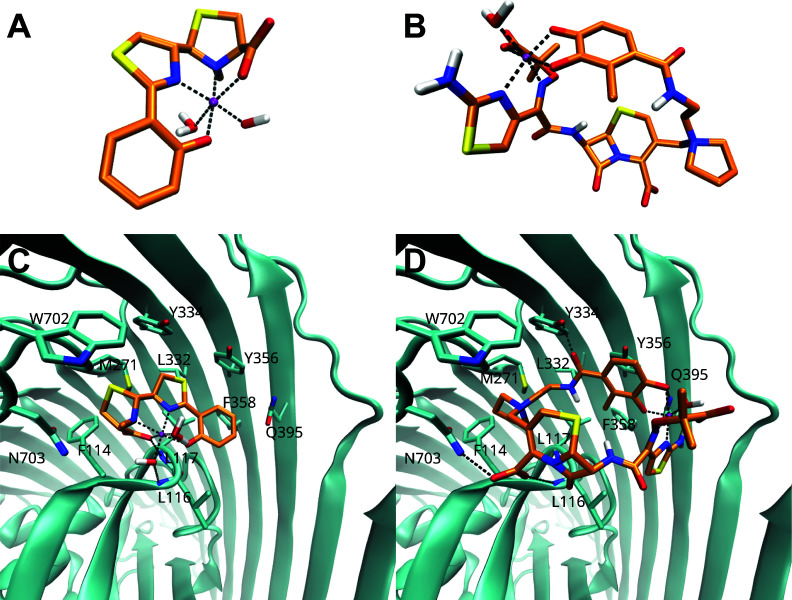
Molecular docking models of pyochelin and cefiderocol with FptA. Modeled structures of (**A**) [Fe(III)-Pyochelin(H2O)OH]^-^ complex and (**B**) [Fe(III)-FDC(H2O)] complex. Docking pose of the (**C**) pyochelin ligand and (**D**) FDC ligand in FptA, showing key interactions with the protein. Carbon atoms of FptA are colored in cyan, and ligand carbon atoms are colored in orange.

Previous studies of clinical isolates of *P. aeruginosa* identified mutations in *fptA* associated with decreased FDC susceptibility after antibiotic exposure both *in vitro* and *in vivo* (6, 11). Overexpression of this receptor sensitized *P. aeruginosa* to FDC (9), suggesting that iron-bound FDC (Fe(III)-FDC) likely utilizes FptA to enter the bacterium. To corroborate that the Fe(III)-FDC complex could bind to FptA, we modeled the complex and performed molecular docking studies. We first validated our docking protocol by reproducing the binding mode of the [Fe(III)–Pyochelin(H₂O)(OH)] complex (Fig. 1A) using its crystallographic structure (PDB 1XKW). The resulting top-ranked pose closely matched the experimentally observed conformation, with a root mean square deviation (RMSD) of 0.53 Å (Fig. S1). We then built *in silico* the complex of the Fe^3+^ ion coordinated to the catechol group of FDC [Fe(III)-FDC(H_2_O)_3_(OH)]^-^. In addition, we constructed two alternative coordination species: one involving the carboxylate group of FDC [Fe(III)–FDC(H₂O)₃] and another involving the methoxy-imino and aminothiazole functionalities of FDC [Fe(III)–FDC(H₂O)]. All three models were used for subsequent docking analyses (Fig. 1B; Fig. S2).

These docking experiments revealed that all complexes were predicted to bind to FptA in the same pocket as Fe(III)-pyochelin. Although the absolute binding energies should be interpreted with caution, the [Fe(III)–FDC(H₂O)] (Fig. 1C and D) species exhibited a binding score comparable to that of the pyochelin complex (ΔΔG of −0.12 kcal/mol). In contrast, the other two coordination modes yielded slightly less favorable scores (ΔΔG of 0.83 kcal/mol and 2.24 kcal/mol), suggesting that the [Fe(III)–FDC(H₂O)] arrangement was the preferred binding mode. This binding pose had similar interactions as the Fe(III)–pyochelin complex (Fig. 1C and D), which consists of a small region with hydrogen bonds to the backbone of residues L116 and L117, and a pocket with numerous hydrophobic/aromatic interactions with residues F114, M271, L332, Y334, Y356, F358, and W702. These predicted interactions suggest that FptA could plausibly function as a receptor for FDC.

### *fptA* inactivation is a first-step mutation toward FDC resistance in PA14

**Fig 2:**
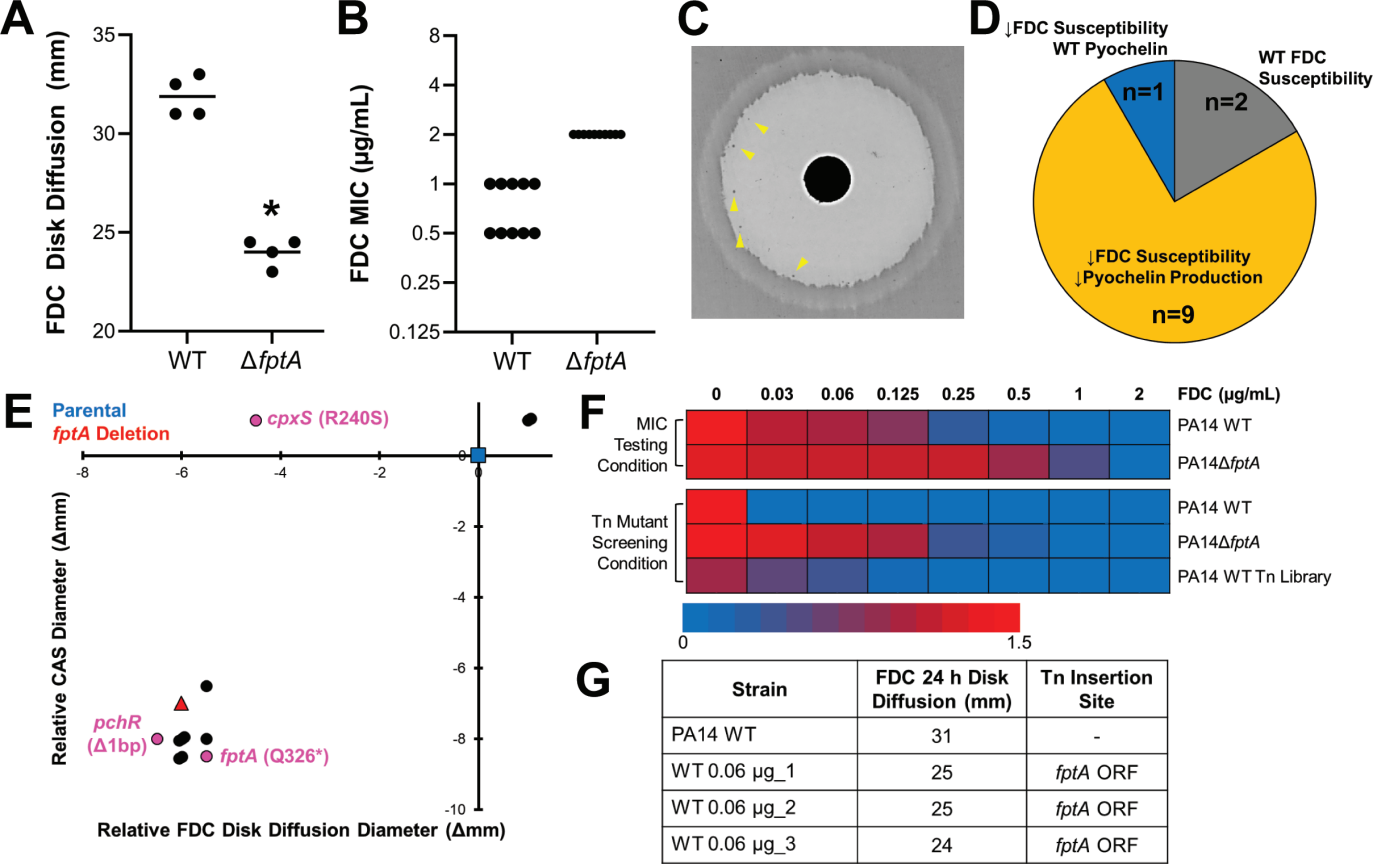
FptA loss is a first-step mutation towards FDC resistance in PA14. (**A, B**) FDC susceptibility for PA14 WT and PA14Δ*fptA*. FDC disk diffusion diameters (**A**) and MICs (**B**) were measured at 24 h. (**C**) FDC disk diffusion assay image for PA14 WT after 48 h growth. Yellow arrows point to inner zone colonies. (**D**) Summary of FDC susceptibility and pyochelin production profiles of FDC inner zone colonies from PA14 WT. (**E**) Changes in FDC susceptibility (disk diffusion diameter) and pyochelin production (Chrome Azurol S assay diameter) for inner zone colonies passaged in drug-free medium, compared to PA14 WT. Blue square: PA14 WT. Red triangle: *PA14*Δ*fptA*. Circles: strains from inner zone colonies. Magenta circles: strains analyzed by whole genome sequencing and their relevant mutations (full list of mutations provided in Table S1). (**F**) PA14 WT, PA14Δ*fptA*, or a library of ~50 K transposon mutants from PA14 WT grown in increasing concentrations of FDC in iron-depleted Mueller-Hinton broth (MIC testing medium) or modified low-iron casamino acids medium (Tn mutants screening condition). Bacterial growth (O.D. 600 nm) was measured after 24 h. (**G**) FDC susceptibility (disk diffusion diameter) and transposon insertion location of three random mutants isolated from the FDC 0.06 µg/mL growth condition. * corresponds to *P* < 0.01 based on a Student’s *t*-test. Each point in (**B**) corresponds to MIC interpreted from a technical replicate from at least three biological replicates.

To confirm whether the loss of the ferripyochelin receptor FptA contributes to FDC resistance in the laboratory reference strain PA14, we generated a *fptA* gene deletion mutant. Loss of *fptA* resulted in decreased susceptibility to FDC, indicated by a significant decrease in the antibiotic disk diffusion diameter and a 2-fold to 4-fold increase in the minimum inhibitory concentration (MIC) (Fig. 2A and B). It was noted that isolated inner-zone colonies emerged within 48 h on disk diffusion assay with wild-type PA14 (Fig. 2C). These colonies were purified, passaged in drug-free medium, and evaluated for FDC susceptibility by disk diffusion testing. Nearly all (*n* = 10/12) inner-zone colonies exhibited decreased susceptibility to FDC (Fig. 2D and E), indicating that these 10 colonies represented spontaneous mutants that acquired a FDC resistance mechanism.

We next screened these spontaneous mutants for siderophore production via a Chrome Azurol S (CAS) activity assay. Removal of iron from CAS by *P. aeruginosa* siderophores causes a chromic shift (blue to yellow), producing a halo around the bacterial lawn (Fig. S3A) (25). On Mueller-Hinton agar, siderophore activity was primarily driven by the smaller and more diffusible siderophore pyochelin, rather than pyoverdine. The pyochelin biosynthetic mutant PA14Δ*pchA* produced a significantly smaller apo-CAS halo compared to wild-type PA14 (Fig. S3A and B). In contrast, the pyoverdine biosynthetic mutant PA14Δ*pvdA* produced a significantly larger halo, probably due to increased pyochelin production in the absence of pyoverdine. As previously shown (26, 27), the inability to produce both siderophores (PA14Δ*pvdA*Δ*pchA*) further prevented iron removal from CAS. Consistent with FptA’s established role in the regulation of pyochelin production by a positive feedback loop (i.e., import of ferripyochelin by FptA promotes siderophore biosynthesis) (23), PA14Δ*fptA* exhibited low siderophore activity on the CAS assay (Fig. S3A and B).

We took advantage of the results of the CAS assay above to screen inner-zone colonies for *fptA* loss-of-function mutations. A total of 9/10 FDC-adapted spontaneous mutants derived from the FDC inner zone exhibited poor pyochelin production (Fig. 2D and E). To confirm the inactivation of *fptA* expression, we performed whole-genome sequencing and mutation analysis. Of the two mutants with decreased pyochelin production sequenced, one harbored a nonsense mutation in *fptA* while the other harbored a frameshift mutation in *pchR*, which encodes the transcriptional regulator for *fptA*. None of these mutants harbored additional mutations previously associated with cefiderocol resistance (Table S1). The one mutant with decreased FDC susceptibility, but wild-type pyochelin production, was found to have a mutation in *cpxS*. This preference for *fptA* inactivation or downregulation in FDC-adapted mutants indicated that FptA loss was likely a first step toward FDC resistance in PA14.

To investigate if *fptA* was also selectively targeted during a single FDC exposure in iron-depleted media, we performed a transposon insertion screen in *P. aeruginosa* PA14, generating a library of ~50 K insertion mutants with the MAR2xT7 mariner transposon (28). Three random mutants were isolated from the batch transposon library culture with the highest concentration of FDC that permitted bacterial growth (Fig. 2F). Transposon insertion sites were identified by Sanger sequencing. All mutants exhibited decreased FDC susceptibility by disk diffusion testing, and all harbored transposon insertions in the *fptA* open reading frame (Fig. 2G), supporting the conclusion that FptA loss was a first-step adaptation to FDC exposure.

While we did not identify pyochelin biosynthetic mutants from the two screens, we posited that disruption of pyochelin production would affect FDC susceptibility by downregulating PchR-dependent transcriptional activation of *fptA* (23). Genetic disruption of pyochelin biosynthesis (Δ*pchA*) decreased FDC susceptibility in both wild-type PA14 and the pyoverdine biosynthetic mutant (PA14Δ*pvdA*) (Fig. S3C). These findings are consistent with a previous study where purified pyochelin sensitized *P. aeruginosa* to FDC and *pchEF* mutants were identified during FDC *in vitro* adaptation (17).

### Second-step mutations following *fptA* inactivation lead to FDC nonsusceptibility in PA14

**Fig 3:**
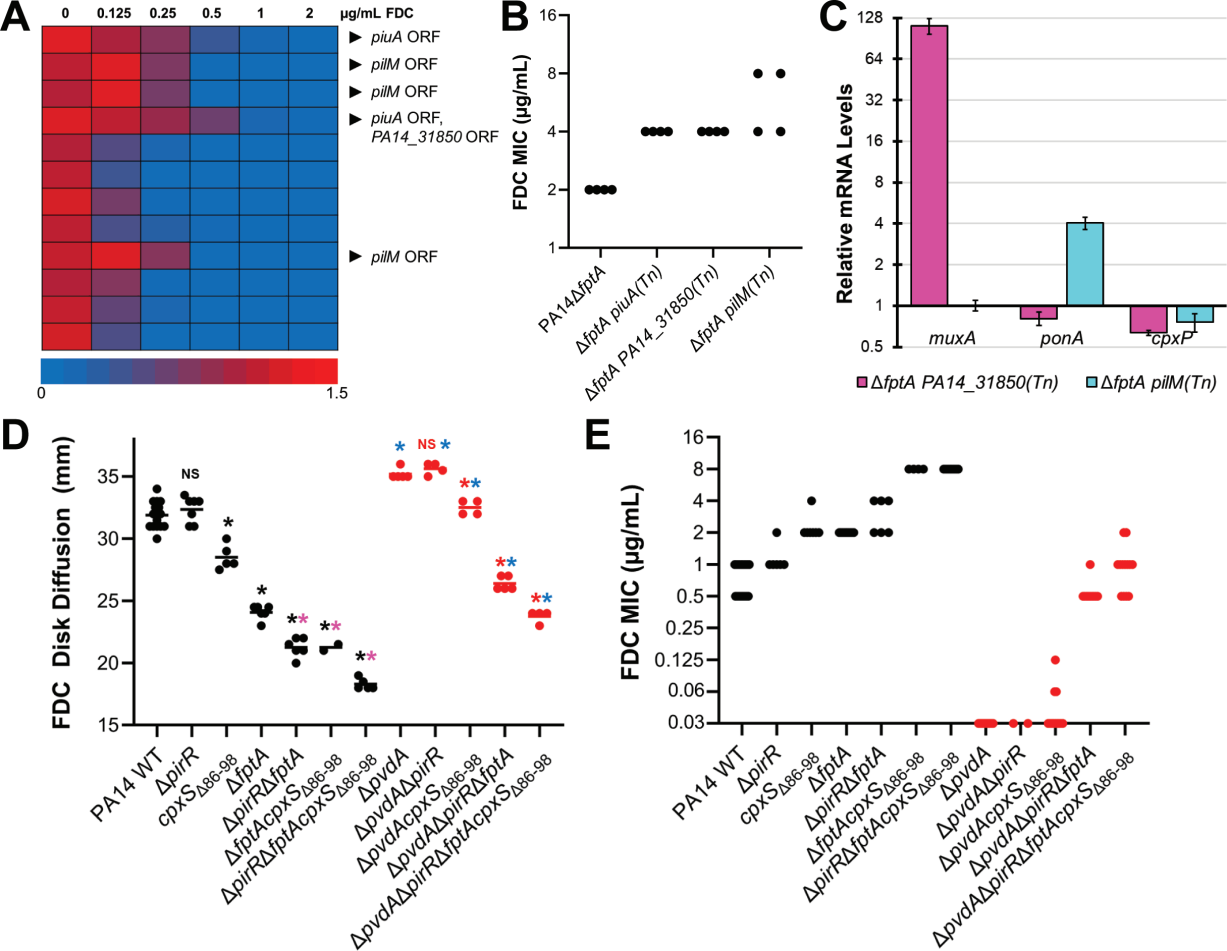
Identification of mutations that confer full FDC nonsusceptibility in PA14. (**A**) Library of ~50 K transposon mutants from PA14Δ*fptA*, split into 12 sublibraries, grown in increasing concentrations of FDC in iron-depleted broth medium. Bacterial growth (O.D. 600 nm) was measured after 24 h. Black arrows point to transposon insertion locations from mutants that were isolated from the condition with the highest concentration of FDC that permitted bacterial growth. (**B**) FDC MICs for PA14Δ*fptA* and three transposon mutants, *piuA (Tn*), *PA14_31850 (Tn*), and *pilM (Tn*), were determined after 24 h growth. (**C**) Relative expression of *muxA*, *ponA*, and *cpxP* (by qRT-PCR) in PA14Δ*fptA* mutants with transposon insertions in *PA14_31850* or *pilM* compared to the PA14Δ*fptA* parental strain. (**D, E**) FDC susceptibility for PA14 mutants. FDC disk diffusion diameters (**D**) and MICs (**E**) were measured at 24 h.* corresponds to *P* < 0.01, NS corresponds to *P* > 0.05 based on a one-way ANOVA with Tukey’s multiple comparisons test. Black: compared to PA14 WT. Magenta: compared to PA14Δ*fptA*. Red: compared to PA14Δ*pvdA*. Blue: comparing Δ*pvdA* mutants to their pyoverdine-producing counterparts. Each point in (**B, E**) corresponds to MIC interpreted from a technical replicate from at least two biological replicates.

Previously, we have identified second-step mutations as an important driver of FDC non-susceptibility in clinical isolates (11). To systematically identify additional mutations associated with loss of FDC susceptibility in the absence of FptA, we performed transposon mutagenesis in PA14Δ*fptA,* generating ~50,000 insertion mutants that were pooled into 12 sub-libraries. Each sub-library was grown in iron-depleted medium with increasing concentrations of FDC, and at least three random mutants were isolated from the culture with the highest concentration of FDC that permitted bacterial growth (Fig. 3A). We identified three distinct mutants with at least a 2-fold increase in the FDC MIC compared to the Δ*fptA* parental strain (Fig. 3B). These insertions were in open reading frames of *piuA* (encoding a catechol siderophore receptor), *PA14_31850* (encoding a hypothetical protein), and *pilM* (type IV pilus biosynthesis gene). While *piuA* encodes a previously identified TonB receptor important for FDC import (9), the latter two genes were not previously identified among FDC or β-lactam resistance genes. However, *PA14_31850* and *pilM* were upstream of *muxA* (multidrug efflux pump gene) and *ponA* (encoding penicillin-binding-protein 1A, PBP1A), respectively. We posited that transposition into the open reading frames of these upstream genes could increase the expression of *muxA* and *ponA* because the gentamicin resistance cassette within the Mar2xT7 mariner transposon lacks a transcriptional terminator sequence, allowing for constitutive activation of downstream sequences from the *aacC1* promoter (28, 29). We measured the mRNA levels of *muxA* and *ponA* by qRT-PCR, which confirmed their overexpression in the transposon mutants (Fig. 3C).

We have previously identified combinations of *pirR* inactivation, *fptA* inactivation, and increased *cpxS* activity in FDC-nonsusceptible clinical isolates, although their specific contribution to the phenotype was not clear (6, 11). Based on these findings and the transposon insertion data, we sought to confirm that introduction of these clinically identified mutations was responsible for the changes in susceptibility to FDC.

Single deletion mutants were constructed for PA14Δ*pirR* in addition to replacing the *cpxS* allele with a variant resulting in the deletion of amino acids 86–98 as identified in a resistant clinical isolate (PA14*cpxS*_Δ86-98_, Fig. S4A) (6). This mutation occurs in the periplasmic sensor domain of CpxS and leads to increased expression of *muxA* and another CpxR-target gene, *cpxP* (Fig. S4B). The FDC disk diffusion diameter and MIC for the PA14Δ*pirR* strain were not significantly different from PA14 wild-type (Fig. 3D and E). The PA14*cpxS*_Δ86-98_ strain showed a decrease in FDC disk diffusion diameter and an increase in MIC of ~2-fold, consistent with altered FDC susceptibility. Multi-gene variants demonstrated further decreases in FDC susceptibility, with the FDC MIC for the PA14Δ*fptA*Δ*pirR* double mutant increasing to near the CLSI breakpoint of 4 μg/mL and the PA14Δ*fptAcpxS*_Δ86-98_ displaying a non-susceptible MIC of 8 μg/mL.

Interestingly, PA14*cpxS*_Δ_*_86-98_* and PA14Δ*fptA* had similar FDC MICs (2 µg/mL) despite our observations that FptA loss was predominantly favored as a first-step adaptation to FDC exposure. To compare the impact of these mutations, we measured the bacterial growth kinetics in increasing concentrations of FDC (Fig. S5). The WT strain exhibited severely delayed growth at FDC concentrations as low as 0.03 µg/mL. Mutations in *pirR* or *cpxS* showed similar growth profiles with an increase in lag phase. In contrast, deletion of *fptA* resulted in less pronounced growth deficits at low FDC concentrations, suggesting improved fitness in the presence of FDC.

While mutations in *cpxS* have been broadly associated with β-lactam resistance (17), mutations in TonB-dependent siderophore receptors or their regulatory elements, such as Δ*fptA* or Δ*pirR*, would be predicted to only affect the import of FDC. To test this hypothesis, we performed antibiotic susceptibility testing for various β-lactams and β-lactam/β-lactamase-inhibitor combinations that are used to treat multidrug-resistant *P. aeruginosa*. Mutations exclusive to siderophore import did not alter the MIC of other β-lactams or combination agents, with no change between PA14 and PA14Δ*fptA*Δ*pirR* double mutant for imipenem-relebactam, ceftazidime-avibactam, ceftolozane-tazobactam, cefepime, or aztreonam (Table 1). Compared to WT PA14, mutants with the *cpxS*_Δ_*_86-98_* allele exhibited a modest ~2-fold increase in the MICs for ceftazidime-avibactam, ceftolozane-tazobactam, and aztreonam, although these MICs remained within the susceptible range. These results demonstrate that while clinically identified FDC resistance mechanisms were sufficient to confer FDC nonsusceptibility in the laboratory strain PA14, the activity of other agents reserved for drug-resistant *P. aeruginosa* infections was minimally impacted.

**TABLE 1:** Cefiderocol resistance mechanisms preserve the activity of other antipseudomonal β-lactam antibiotics^*a*^

MICs (μg/mL)	Imipenem-relebactam		Ceftazidime-avibactam		Ceftolozane-tazobactam		Cefepime		Aztreonam	
	Range	Mode	Range	Mode	Range	Mode	Range	Mode	Range	Mode
PA14 WT	0.38–0.5	0.5	0.5–1	0.5	0.38–0.5	0.5	1	1	2–3	3
PA14Δ*pirR*Δ*fptA*	0.38–0.5	0.5	0.5–0.75	0.5	0.38	0.38	1–1.5	1	3	3
PA14*cpxS*_Δ86-98_	0.38–0.5	0.38	1.5	1.5	1–1.5	1.5	1–1.5	1.5	6	6
PA14Δ*pirR*Δ*fptAcpxS*_Δ86-98_	0.38–0.5	0.38	1–1.5	1	1–2	1.5	1	1	4–6	6

^*a*^ MICs for antipseudomonal β-lactams and β-lactam/β-lactamase inhibitor combinations for PA14 wild type (WT), PA14Δ*pirR*Δ*fptA*, PA14*cpxS*_Δ_*_86-98_*, and PA14Δ*pirR*Δ*fptAcpxS*_Δ_*_86-98_*. MICs were determined by the gradient diffusion strip test on Mueller-Hinton agar after 18 h.

### Impact of pyoverdine production on FDC susceptibility

In addition to decreased import via loss of siderophore uptake receptors, pyoverdine production has been implicated in FDC resistance in *P. aeruginosa*. Due to its exceptionally high affinity for ferric iron, pyoverdine has been shown to chelate iron away from FDC (17), which would prevent the antibiotic from utilizing siderophore uptake receptors. Several reports have demonstrated a positive correlation between pyoverdine production and FDC resistance in *P. aeruginosa* clinical isolates (17, 30). To determine the extent of pyoverdine’s role in the FDC-nonsusceptible mutant PA14Δ*pirR*Δ*fptAcpxS*_Δ_*_86-98_*, we introduced these mutations into PA14Δ*pvdA*, a pyoverdine biosynthetic mutant. In the absence of pyoverdine, the three FDC resistance mechanisms were not sufficient for full nonsusceptibility (MIC = 1 µg/mL) (Fig. 3E). We observed similar ~8-fold decreases in FDC MICs and significant increases in FDC disk diffusion diameters for each intermediate mutant we generated in the Δ*pvdA* background (Fig. 3D and E), supporting the role of pyoverdine production in FDC resistance.

### Loss of FptA confers a fitness cost in a pyoverdine biosynthetic mutant

**Fig 4:**
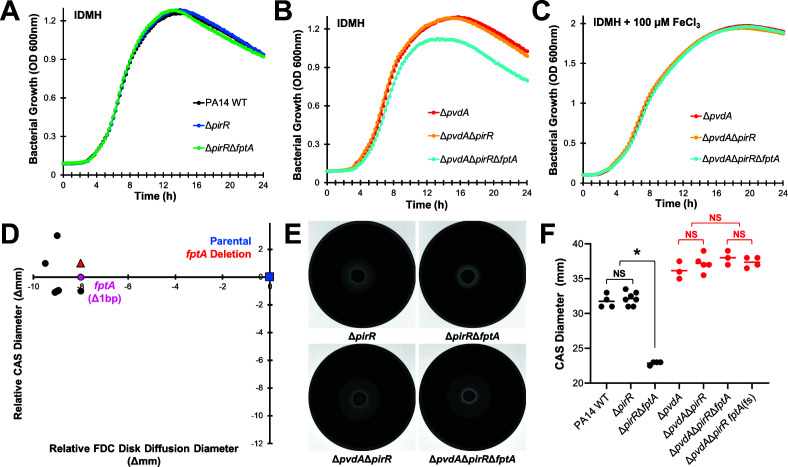
Inactivation of *fptA* confers a fitness cost in a pyoverdine biosynthetic mutant. (**A–C**) Bacterial growth (O.D. 600 nm) was measured every 30 min for 24 h in iron-depleted Mueller-Hinton broth with (**A, B**) or without (**C**) ferric iron (100 µM FeCl_3_) supplementation for PA14 mutants. Each point represents the average across four biological replicates. (**D**) Changes in FDC susceptibility (disk diffusion diameter) and pyochelin production (Chrome Azurol S assay diameter) for inner zone colonies isolated from a FDC disk diffusion assay for PA14Δ*pvdA*Δ*pirR*, compared to parental strain control. Blue square: PA14Δ*pvdA*Δ*pirR*. Red triangle: PA14Δ*pvdA*Δ*pirR*Δ*fptA*. Circles: strains from inner zone colonies. Magenta circle: strain analyzed by whole genome sequencing, relevant mutation indicated (full list of mutations provided in Table S1). (**E**) Visualization of pyochelin production by PA14 mutants on Mueller-Hinton agar supplemented with CAS-Fe^3+^ after 48 h. (**F**) Diameter of the apo-CAS halo that represents pyochelin production. * corresponds to *P* < 0.01, NS corresponds to *P* > 0.05 based on a one-way ANOVA with Tukey’s multiple comparisons test.

Based on previous studies (31–33), we predicted that the inability to uptake ferripyochelin in the absence of pyoverdine production would substantially disrupt bacterial iron homeostasis, conferring a fitness cost in iron-restricted media. As confirmation, we measured bacterial growth kinetics in IDMH with or without ferric iron supplementation (100 µM FeCl_3_) for pyoverdine-producing (WT PA14, PA14Δ*pirR*, and PA14Δ*pirR*Δ*fptA*) and non-producing (PA14Δ*pvdA*, PA14Δ*pvdA*Δ*pirR*, and PA14Δ*pvdA*Δ*pirR*Δ*fptA*) mutants. In pyoverdine-producing strains, inactivation of *fptA* did not affect bacterial growth in IDMH (Fig. 4A). In the Δ*pvdA* mutants, loss of *fptA* hampered bacterial growth (Fig. 4B), with significantly lower densities (O.D. 600 nm) throughout the growth curve (Table S2). However, these growth defects were fully rescued with ferric iron supplementation (Fig. 4C; Table S2), demonstrating that iron starvation impeded bacterial growth in the absence of pyoverdine production and ferripyochelin uptake. These differences in bacterial growth were more noticeable under severe iron starvation in the presence of the ferric iron chelator ethylenediamine-N,N′-bis(2-hydroxyphenylacetic acid) (EDDHA). Consistent with previous reports (34, 35), pyoverdine production was required for bacterial growth in EDDHA-supplemented media (Fig. S6A), but we observed dose-dependent growth inhibition in the pyoverdine biosynthetic mutant (PA14Δ*pvdA* and PA14Δ*pvdA*Δ*pirR*) at EDDHA concentrations below 0.5 mg/L (Fig. S6B and C). Under these conditions, we observed further growth inhibition by the inactivation of *fptA* (PA14Δ*pvdA*Δ*pirR*Δ*fptA*) (Fig. S6D).

Based on these findings, we examined whether FptA loss remained favored as an adaptation strategy toward FDC resistance in the pyoverdine biosynthetic mutant PA14Δ*pvdA*Δ*pirR*. This genetic background was selected because *fptA* inactivation had a more profound effect on FDC susceptibility in the absence of *pirR* (Fig. 3D). We screened inner-zone colonies that emerged within 48 h from a FDC disk diffusion assay, verifying decreased FDC susceptibility for the spontaneous mutants by disk diffusion testing and reduced pyochelin production using a CAS siderophore activity assay (Fig. 4D). None of the FDC-adapted mutants exhibited decreased pyochelin production. We performed whole-genome sequencing and mutation analysis for one randomly selected mutant. Unexpectedly, this mutant harbored a *fptA* frameshift mutation with no other mutations previously associated with FDC resistance (Table S1). Measuring pyochelin production in the PA14Δ*pvdA*Δ*pirR*Δ*fptA* deletion mutant by CAS assay confirmed that loss of the ferripyochelin receptor in the absence of pyoverdine did not downregulate pyochelin biosynthesis (Fig. 4E and F), possibly due to a severe iron-starvation response. Without functional FptA, this mutant would not have been able to effectively utilize pyochelin, which could have also contributed to the growth defects we observed. Importantly, while we were not able to fully screen FDC-adapted, pyoverdine biosynthetic mutants for the loss of *fptA* expression or function, the selection of the *fptA* frameshift mutant suggests that inactivation of this outer membrane receptor remained one of the major mechanisms of FDC resistance despite the fitness cost.

### Fitness costs drive *fptA* allele frequency in mixed populations

**Fig 5:**
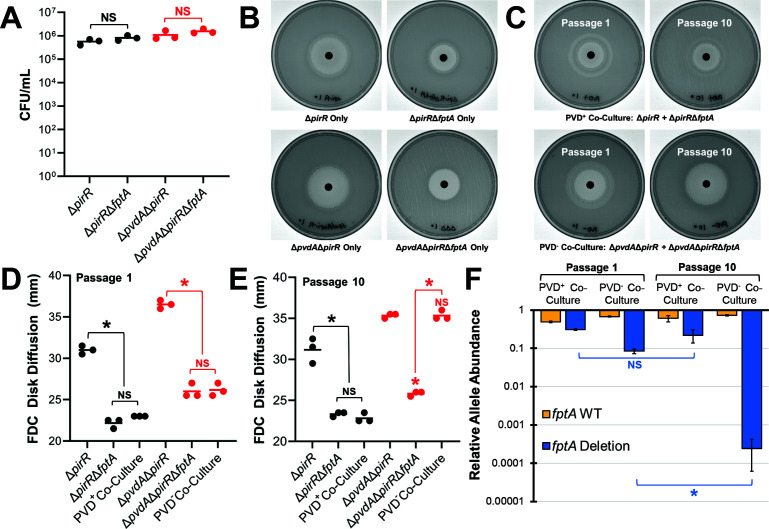
Δ*fptA* is not stably maintained in a pyoverdine biosynthetic mutant. (**A**) Initial inoculum of PA14 mutants in the serial passage experiment. (**B**) Images of FDC disk diffusion testing plates for monocultures of PA14 mutants. (**C**) Images of FDC disk diffusion testing plates for co-cultures of PA14Δ*pirR* and PA14Δ*pirR*Δ*fptA* (top) or PA14Δ*pvdA*Δ*pirR* and PA14Δ*pvdA*Δ*pirR*Δ*fptA* (bottom) passaged in iron-depleted Mueller-Hinton broth for 1 or 10 days. (**D, E**) Quantification of FDC disk diffusion diameters from assays performed after the first (**D**) or tenth passage (**E**). (**F**) Quantification of the *fptA* WT and Δ*fptA* alleles in the co-cultures after the first or tenth passage via qPCR using allelic-specific primers. * corresponds to *P* < 0.01, NS corresponds to *P* > 0.05 based on a one-way ANOVA with Sidak’s (**A, F**) or Tukey’s (**D, E**) multiple comparisons test.

Finally, we hypothesized that in the absence of pyoverdine, acquisition of *fptA* mutations could result in unstable subpopulations with reduced FDC susceptibility. Without selective pressure from FDC to offset fitness costs, these mutations would not be maintained at stable frequencies across the population. As proof of concept, we passaged inoculum-controlled (identical starting inoculum of ~10^6^ CFU/mL, Fig. 5A) co-cultures of isogenic *fptA*^+^ and *fptA*^-^ strains in the presence (PA14Δ*pirR* and PA14Δ*pirR*Δ*fptA*) or the absence (PA14Δ*pvdA*Δ*pirR* and PA14Δ*pvdA*Δ*pirR*Δ*fptA*) of pyoverdine production. Throughout the 10-day experiment, FDC susceptibility of the pyoverdine-producing co-cultures remained stable, with no significant changes in FDC disk diffusion diameters between days 1 and 10 of passaging (Fig. 5B through E). However, co-cultures of the pyoverdine biosynthetic mutants exhibited a gradual sensitization of the population to FDC with a visible dwindling of the Δ*fptA* subpopulation, which was no longer observable by the end of the experiment (Fig. 5B through E; Fig. S7). These observations were validated by qPCR analysis, which indicated a significant, 3-log reduction in the frequency of the Δ*fptA* allele between the first and tenth passage for the pyoverdine-null cultures (Fig. 5F). In contrast, the Δ*fptA* allele remained stable for the pyoverdine-producing cultures. These results suggest that for *P. aeruginosa* strains that do not produce pyoverdine, the selection of *fptA* mutations may rely on continuous selective pressure from the antibiotic and represent an unstable subpopulation with reduced FDC susceptibility.

## DISCUSSION

In this study, we used the laboratory strain PA14 to characterize the stepwise events leading to FDC non-susceptibility. We found that loss of function of the ferripyochelin receptor FptA was a frequent first step to reduced susceptibility on both Mueller-Hinton agar and in iron-depleted broth medium. This is different from a previous study that examined the laboratory strain PAO1, where the inactivation of the catechol siderophore receptor *piuA* appeared to be the first-step mutation toward FDC resistance (18). In another PAO1-based study, disruption of *fptA* by transposon insertion did not affect the FDC MIC, while disruption of *piuA* increased the MIC by 16-fold (5). Nevertheless, *fptA* mutations have been identified in several long-term adaptive evolution experiments in PAO1 (17, 18). Observations in other clinical and laboratory strains suggest that inactivation of TonB-dependent receptors may have disparate impacts on FDC import, depending on the *P. aeruginosa* strain (36). While some strains exhibited loss of *piuA* under *in vivo* or *in vitro* FDC-selective pressure (37, 38), others exhibited loss of *fptA* (6, 11, 39), both (11, 18), or neither (37). A systematic transcriptomic and proteomic study of these strains under iron-restrictive conditions would be required to determine whether there are biological differences that determine which receptors are important for FDC entry. It is also important to note that expression of these siderophore uptake receptors *in vitro* (e.g., iron-depleted Mueller-Hinton broth) may not represent physiological conditions. For instance, one study recently demonstrated that *P. aeruginosa* acquires distinct FDC resistance mechanisms in synthetic cystic fibrosis sputum medium and synthetic urine medium (17). Further considerations would be needed to model *in vivo* conditions, where host iron-sequestering proteins, such as transferrin, and alternative iron sources, such as heme are present.

In PA14, single mutations of *fptA* were not sufficient to confer a resistant phenotype, and we identified a number of second-step mutations that work in concert with decreased uptake via loss of FptA. Several of these mechanisms have been previously described, including upregulation of genes in the *cpxS* regulon (17) and inactivation of the siderophore transporter encoded by *piuA* (5, 9, 12). However, we also identified a transposon insertion mutant that led to increased expression of the *ponA* gene encoding PBP1A. Increased levels of the bifunctional peptidoglycan transpeptidase/glycosyltransferase PBP1A may partially compensate for the inhibition of PBP3-transpeptidase activity by FDC (40).

While previous studies have identified mutations in *pirR*, *cpxS*, and *fptA* in association with FDC resistance, these mutations were frequently found in conjunction with multiple other changes, making discerning their specific contribution difficult (17, 18). This study compared the direct impact of these mutations alone and in combination using allelic exchange in a laboratory *P. aeruginosa* background, confirmed via whole genome sequencing. Individually, loss of FptA and activation of CpxS both led to a shift in FDC MIC, and together, these changes were sufficient to confer an FDC non-susceptible phenotype. In contrast, loss of PirR showed minimal changes in FDC disk diffusion diameter or MIC, suggesting that additional differences in growth environment or strain background are needed for the loss of PirR to exert an effect. Importantly, we found that mutations arising from FDC exposure in PA14 display limited cross-resistance with other anti-pseudomonal β-lactam antibiotics, including imipenem-relebactam, ceftazidime-avibactam, and ceftolozane-tazobactam. This contrasts with the potential for the selection of cross-resistance to FDC that has been observed after ceftolozane-tazobactam or ceftazidime-avibactam exposure in the literature (20, 21, 41). While further study is needed, this lack of cross-resistance after FDC exposure could suggest the use of FDC up front against select DTR-*P. aeruginosa* isolates, rather than as a salvage regimen.

Interestingly, overexpression of the *muxABC-opmB* operon via *cpxS*_Δ_*_86-98_* reduced bacterial susceptibility to FDC even in the absence of pyoverdine (in PA14Δ*pvdAcpxS*_Δ_*_86-98_* and PA14Δ*pvdA*Δ*pirR*Δ*fptAcpxS*_Δ_*_86-98_* backgrounds). This contrasts with a previous study that hypothesized that mutations in *cpxS* contribute to FDC resistance in a pyoverdine-dependent manner, where MuxABC-OpmB is predicted to have a direct role in pyoverdine secretion (17). In iron-depleted Mueller-Hinton broth (IDMH), the *cpxS*_Δ_*_86-98_* mutation did not affect pyoverdine production (Fig. S4C), suggesting that increased drug efflux may be responsible for the decrease in FDC susceptibility observed in our study.

Finally, we determined the fitness of *fptA* deletion mutants *in vitro* across both iron-limited conditions and in strains deficient in pyoverdine production. Our results suggest that pyoverdine production mitigates the impact of FptA loss, likely by providing an alternative pathway for iron acquisition. The concomitant production of pyoverdine with loss of ferripyochelin import may synergize in maintaining clones with reduced FDC susceptibility in the population. Indeed, in a pyoverdine biosynthetic mutant background, passage in the absence of FDC led to a decrease in the frequency of the *fptA* deletion allele. The resulting population demonstrated a susceptible disk diffusion diameter despite a low frequency of *fptA* mutants in the population, suggesting a possible mechanism for the emergence of FDC heteroresistance in *P. aeruginosa* strains that have lost the ability to produce pyoverdine, commonly found in patients with cystic fibrosis (42–45).

In summary, we evaluated the major contributions of FptA to FDC susceptibility and fitness in the laboratory strain PA14. Loss of FptA along with second-step mutations in the CpxS histidine kinase, TonB receptors, and increased expression of PBP1A were found to contribute to decreased FDC activity. Strains with deletion of *fptA* demonstrated fitness defects in the absence of pyoverdine production, which contributed to a decrease in the allele frequencies of the deletion mutant in a co-culture experiment. Further work is needed to explore the intersection of FDC resistance-associated fitness costs and the heteroresistant phenotype.

## MATERIALS AND METHODS

### Molecular docking

Molecular docking studies were performed using Autodock GPU (46, 47). Grid maps of 70 × 70 × 70 points were used, with a grid spacing of 0.375 Å, centered on the Fe atom of the original Fe(III)-Pyochelin complex in PDB 1XKW (48). For each docking calculation, 500 different docking runs were performed, which were clustered using an RMSD cutoff of 2 Å. All the ligand complexes were modeled and optimized using Gaussian 16 (49), using B3LYP/6-31G** for C, N, O, S, and H atoms, and B3LYP/LANL2DZ for Cl and Fe atoms. Ligand RESP charges were calculated using Multiwfn (50, 51).

### Bacterial strains and allelic exchange mutagenesis

The Δ*pirR* (Δ444 bp coding), Δ*fptA* (Δ1,163 bp coding), and *cpxS*_Δ86-98_ mutations were introduced into *P. aeruginosa* PA14 (52) and pyoverdine biosynthetic mutant PA14Δ*pvdA* (53) using allelic exchange mutagenesis by the pEXG2 vector as previously described (54). In brief, regions upstream and downstream (~600 bp) of the gene deletion site were amplified by polymerase chain reaction (PCR) and cloned into linearized pEXG2 (digested by SacI and XbaI [New England Biolabs, NEB]) via Gibson assembly (NEB). The assembled vector was transformed into competent *Escherichia coli* DH5α cells (NEB) by selecting for gentamicin resistance. The tandem insertion of the upstream and downstream regions into the plasmid was verified by PCR and Sanger sequencing (Azenta Genewiz). The vector was then transformed into competent *E. coli* SM10 conjugal donor cells. PA14 and SM10 were mated on Tryptic Soy Agar (TSA) (BD), and single-crossover merodiploid mutants were selected on TSA supplemented with 30 µg/mL gentamicin (Millipore Sigma) and 10 µg/mL triclosan (Millipore Sigma). Merodiploid colonies were grown in antibiotic-free LB broth (BD) and counter-selected for the *sacB* gene on no-sodium LB agar (NSLB) with sucrose (5 g/L yeast extract [BD], 10 g/L tryptone [Gibco], 20 g/L agar [BD], 20% wt/vol sucrose [Fisher Scientific], 10 µg/mL triclosan). PA14 mutants were verified by PCR to confirm the gene deletion and by Illumina sequencing to confirm the absence of additional extraneous mutations. All primer sequences are available in Table S3.

### FDC susceptibility testing

Iron-depleted Mueller-Hinton II broth (IDMH) was prepared according to Clinical and Laboratory Standards Institute guidelines (CLSI M100 35th Ed.) using Chelex 100 resin (BioRad) to adjust iron concentrations to < 0.03 mg/L (55). For FDC broth microdilution testing, *P. aeruginosa* overnight cultures (16–20 h) were grown in IDMH. In total, 200-fold dilution of a 0.5 McFarland standard (O.D. 600 nm 0.08–0.1, corresponding to ~10^8^ CFU/mL) in IDMH was used as the starting inoculum. The assay was performed in 96-well round-bottom plates (Corning) (150 µL/well) for FDC (Shionogi) concentrations 0.03–32 µg/mL. MICs were interpreted visually after 24 h incubation at 37°C.

FDC disk diffusion testing was performed on Mueller-Hinton II agar using 30 µg FDC disks (Hardy Diagnostics) and 0.5 McFarland standard inoculum from IDMH overnight cultures. Zones of inhibition were measured after 24 h incubation at 37°C.

### Chrome azurol S (CAS) siderophore production assay

A modified CAS agar medium was prepared using previously established methods (56). The CAS reagent solution was prepared in 250 mL ddH_2_O (1 mM Chrome Azurol S (Honeywell Fluka), 0.1 mM FeCl_3_, and 2 mM hexadecyltrimethylammonium bromide (HDTMA) (Acros Organics)) and autoclaved in a plastic container. In total, 750 mL Mueller-Hinton II agar (BD) (concentration adjusted for 1 L final volume) was prepared and autoclaved in a separate plastic container, combined with 250 mL CAS reagent solution, and supplemented with 10 µg/mL triclosan. CAS assay was performed by spotting 10 µL of 0.5 McFarland standard inoculum in IDMH onto a 10 cm CAS agar plate. Diameter of the apo-CAS halo around the bacterial lawn was measured after 24 h and 48 h incubation at 37°C.

### Next-generation sequencing and mutation analysis

Single-nucleotide polymorphisms, insertions, and deletions in PA14 gene deletion mutants or FDC-adapted strains were identified by whole-genome sequencing, as previously described (57). Briefly, strains were grown at 37°C in a shaking incubator for 3–6 h, pelleted, and genomic DNA was extracted using the DNeasy blood and tissue kit (Qiagen). Whole genome sequencing was carried out on a NextSeq2000 platform (Illumina) with 2 × 300 paired-end reads. Mutations were identified by aligning short-read sequences to the UCBPP-PA14 reference genome (NC_008463.1) using Bowtie2 version 2.4.5 built under the *breseq* pipeline (version 0.39.0) (58).

### Transposon mutagenesis

Transposon mutagenesis was performed using an *E. coli* SM10 conjugal donor strain carrying the pMAR2xT7 plasmid that expresses the Mariner transposon (28). PA14 wild-type or PA14Δ*fptA* was mated with *E. coli* SM10 on TSA, and transposon mutants were selected on TSA supplemented with 30 µg/mL gentamicin and 10 µg/mL triclosan. For PA14 WT, ~50 K transposon mutants were pooled into one library. For PA14Δ*fptA*, ~50K transposon mutants were pooled into 12 sublibraries. Transposon mutant libraries were stored at −80°C before use.

To screen transposon mutants for decreased FDC susceptibility, libraries were thawed and grown in M9 low-iron casamino acids medium (11.28 g/L Difco 5× M9 Salts [BD], 17.5 g/L Bacto Casamino Acids, low sodium chloride and iron concentrations [Gibco], 0.5 mM MgCl_2_, 0.5 mM CaCl_2_) and treated with 0.03–32 µg/mL FDC (1:1 M9 low-iron medium and IDMH) in a 96-well round-bottom plate for 24 h at 37°C. Mutants were isolated from the condition with the highest concentration of FDC that permitted bacterial growth. The transposon insertion sites in these mutants were identified by Sanger sequencing using arbitrary and transposon-specific primers as previously described (28).

### Quantitative real-time PCR (qRT-PCR)

qRT-PCR was performed to measure the gene expression (mRNA) levels for CpxR-regulated genes (*muxA* and *cpxP*), genes encoding TonB-dependent siderophore receptors (*piuA* and *pirA*), and genes downstream of select transposon insertion mutants (*ponA* and *muxA*). *P. aeruginosa* was grown in M9 low-iron casamino acids medium in a 50 mL conical tube (10 mL) for 4 h at 37°C with vigorous shaking with a starting inoculum of ~5 × 10^7^ CFU/mL. RNA isolation was performed as previously described (59) using Trizol reagent (Invitrogen) according to manufacturer’s protocols. DNA contaminants were removed from the purified extract by TURBO DNase (Invitrogen), and reverse transcription was performed using a LunaScript RT Supermix (NEB). qRT-PCR was performed using PowerUp SYBR Green Master Mix (Applied Biosystems) in a CFX Opus 96 Real-Time PCR System (BioRad). Relative gene expression was calculated using a ΔΔCt method based on the values for PA14 WT and using *gyrB* as the housekeeping gene.

### Bacterial growth and pyoverdine production measurement

Bacterial growth (O.D. 600 nm) was measured using a Synergy H1 multimode microplate reader (BioTek) using a 96-well round-bottom plate (with or without FDC) prepared as above. For growth curves, absorbance was measured every 30 min for 24 h with continuous incubation at 37°C. Pyoverdine production was measured from IDMH overnight cultures by fluorescence (Ex. 405 nm; Em. 460 nm) in a 96-well black, clear flat-bottom plate (Corning).

### FptA^+^/FptA^-^ mixed population passaging

FptA^+^ (WT) and FptA^-^ (Δ*fptA*) mutants were generated for pyoverdine-producing (PA14Δ*pirR*) and non-producing (PA14Δ*pvdA*Δ*pirR*) strain backgrounds. FptA^+^/FptA^-^ co-cultures were mixed at a 1:1 ratio (~10^6^ CFU/mL each) in 2 mL IDMH and passaged in a 15 mL conical tube every 24 h for 10 days. At each passage, the co-culture was diluted 10,000-fold in IDMH, and FDC disk diffusion testing was performed. Cells were harvested at the beginning (day 1) and end (day 10) of the experiment for allelic abundance analysis.

The relative abundance of each *fptA* allele in the co-culture was quantified by qPCR. Primers were designed for ~100 bp amplification within the gene deletion region (WT-specific primers), upstream and downstream regions flanking the deletion site (Δ*fptA*-specific primers), or within the downstream region shared by both WT and Δ*fptA* strains (nonspecific primers). Genomic DNA from the co-cultures was extracted using a DNeasy UltraClean Microbial Kit (Qiagen) and adjusted to a final concentration of 100 ng/µL using a Nanodrop One spectrophotometer (ThermoFisher Scientific). qPCR reactions were performed using a PowerUp SYBR Green Master Mix in a CFX Opus 96 Real-Time PCR System. The relative abundance of the WT *fptA* and Δ*fptA* alleles in the co-culture was calculated using a ΔCt method:

Allelic abundance = 2^-ΔCt^, where ΔCt = [Ct for WT or Δ*fptA*-specific] – [Ct for nonspecific]

### Statistical analysis

Student’s *t*-test and one-way ANOVA with multiple comparisons tests were performed using GraphPad Prism 10.

## Data Availability

The whole genome sequencing data is available at the National Center for Biotechnology Information (NCBI) website, Bioproject accession number PRJNA1316631.

## SUPPLEMENTAL MATERIAL

Supplemental figures aac.01410-25-s0001.pdf Fig. S1 to S7.

Table S1 aac.01410-25-s0002.xlsx Genome sequencing of mutants.

Table S2 aac.01410-25-s0003.xlsx Statistical comparisons.

Table S3 aac.01410-25-s0004.xlsx Primers used in study.

ASM does not own the copyrights to Supplemental Material that may be linked to, or accessed through, an article. The authors have granted ASM a non-exclusive, world-wide license to publish the Supplemental Material files. Please contact the corresponding author directly for reuse.
