# Supplementary material for: Loss of the ferripyochelin receptor FptA drives reduced cefiderocol susceptibility and impairs fitness in Pseudomonas aeruginosa PA14
Source: Antimicrob Agents Chemother. 2026 Feb 12;70(3):e01410-25. doi: 10.1128/aac.01410-25 (PMC12959141; doi:10.1128/aac.01410-25)
Supplement: Supplemental figures — Fig. S1 to S7. [file aac.01410-25-s0001.pdf]

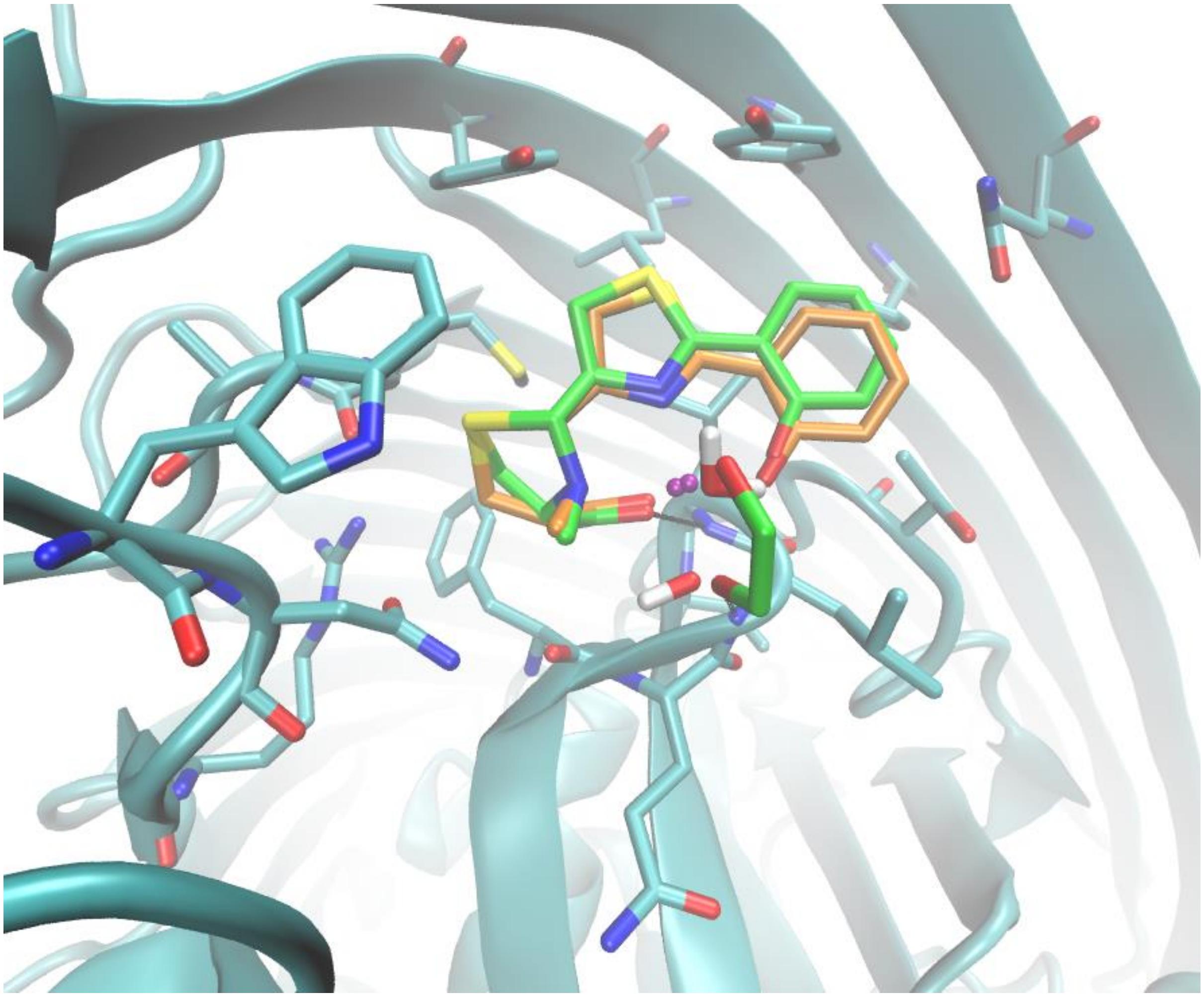

Figure S1. Superposition of the crystallographic structure 1XKW and the docking simulation of the Fe(III)-Pyochelin complex. Carbon atoms are colored in cyan for the protein, green for the ligand's crystallographic conformation, and orange for the ligand's docking pose.

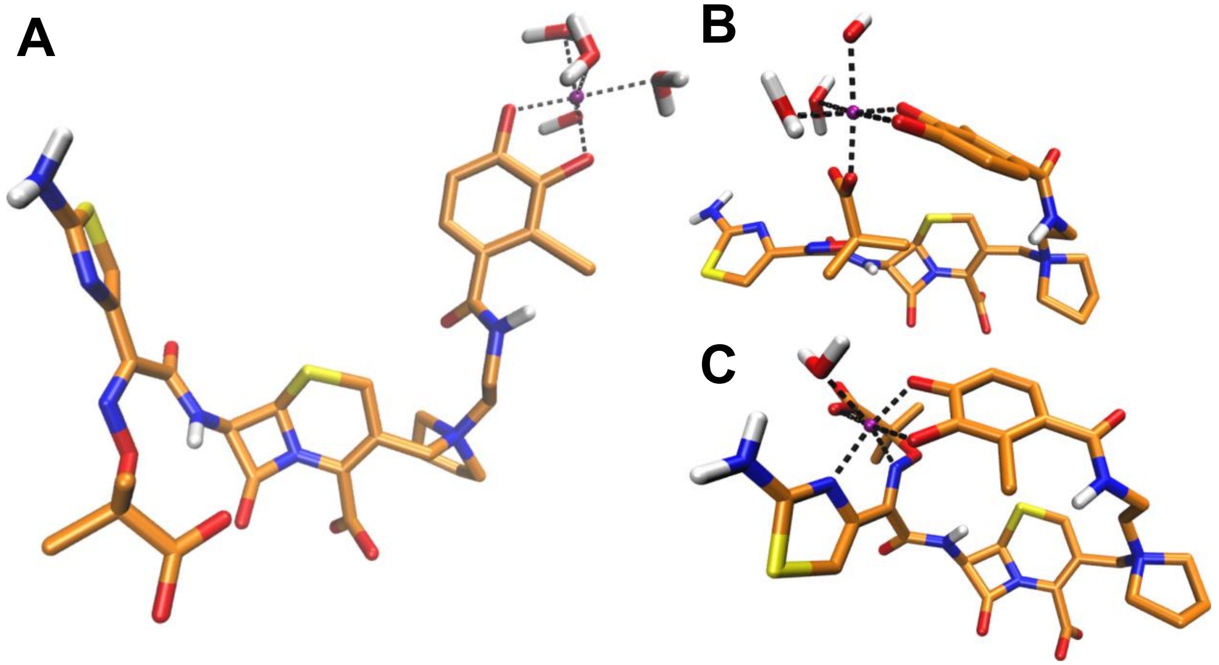

Figure S2. Fe(III)-FDC models built in-silico. (A) [Fe(III)-FDC(H2O)3OH]$^{-}$ **(B)** [Fe(III)–FDC(H_2O)$_3$] **(C)** [Fe(III)–FDC(H_2O)]

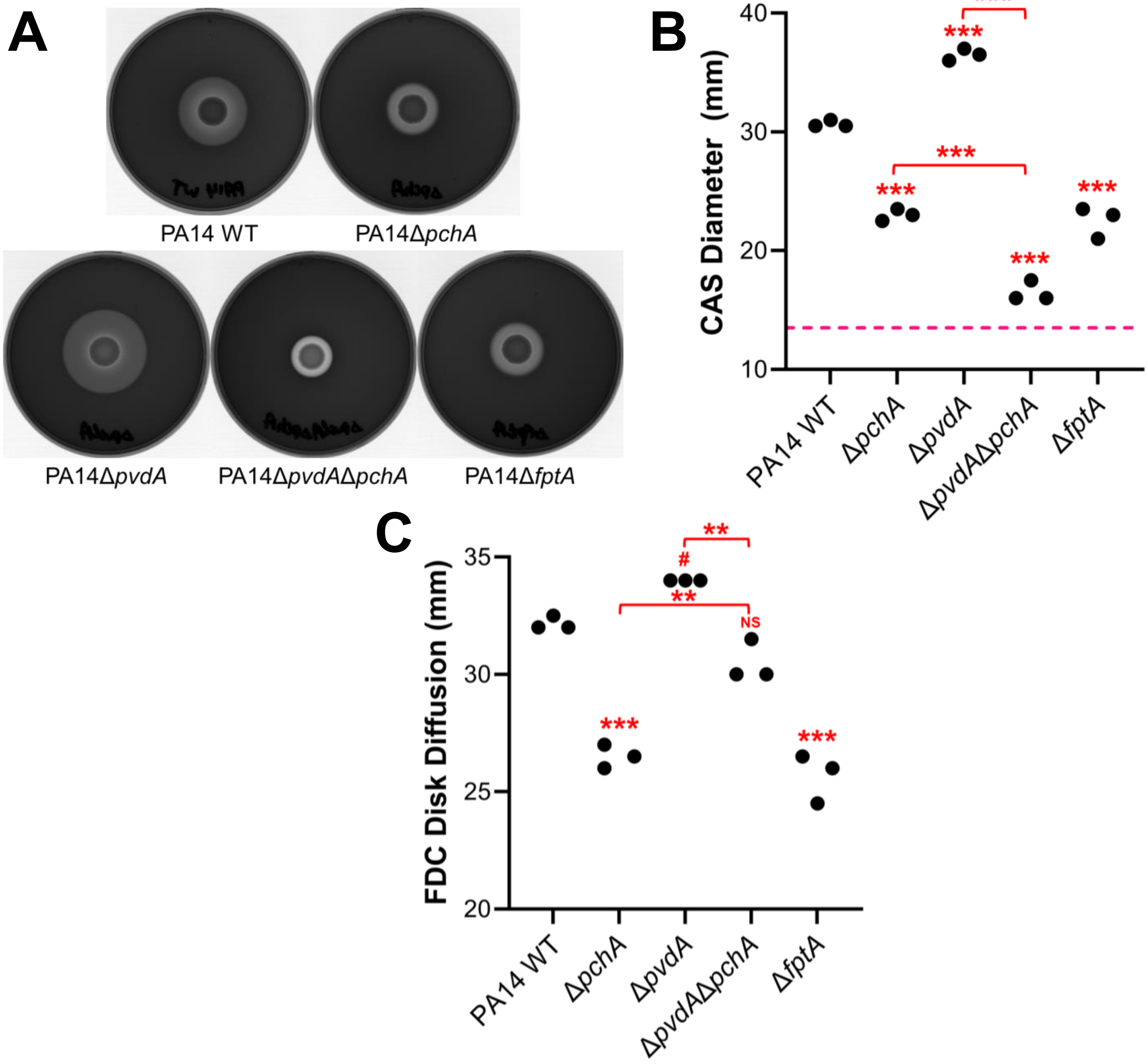

Figure S3. Chrome Azurol S (CAS) siderophore activity assay measures pyochelin production on Mueller-Hinton agar. (A) Visualization of siderophore production by PA14 mutants on Mueller-Hinton agar supplemented with CAS-Fe^{3+} after 48 h. **(B)** Quantification of siderophore production using the diameter of the apo-CAS halo. Dotted line represents limit the lower limit of detection for the assay (size of the bacterial lawn). **(C)** FDC disk diffusion diameters measured at 24 h for PA14 mutants. NS corresponds to $p > 0.05$, # corresponds to $p < 0.05$, ** corresponds to $p < 0.001$, *** corresponds to $p < 0.0001$ based on a one-way ANOVA with Tukey's multiple comparisons test.

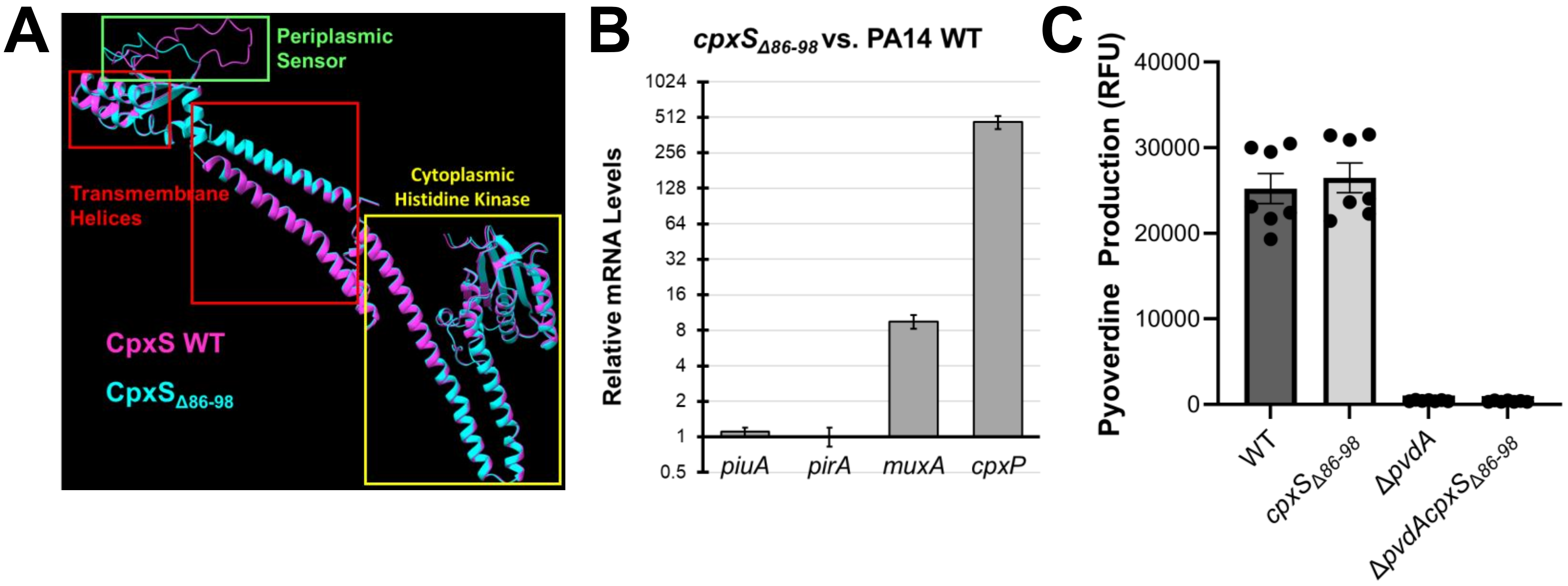

Figure S4. Mutation in *cpxS* leads to *muxA* overexpression. (A) Alpha-fold protein structure prediction and superimposition ("Matchmaker") for CpxS from PA14 WT and PA14*cpxS*$_{\Delta 86\text{-}98}$. Structures were generated by ChimeraX. **(B)** Expression of *piuA*, *pirA*, *muxA*, and *cpxP* (by qRT-PCR) in PA14*cpxS*$_{\Delta 86\text{-}98}$ compared to PA14 WT. **(C)** Pyoverdine production (measured in fluorescence - Ex. 405 nm; Em. 460 nm) in iron-depleted Mueller-Hinton broth by PA14 mutants.

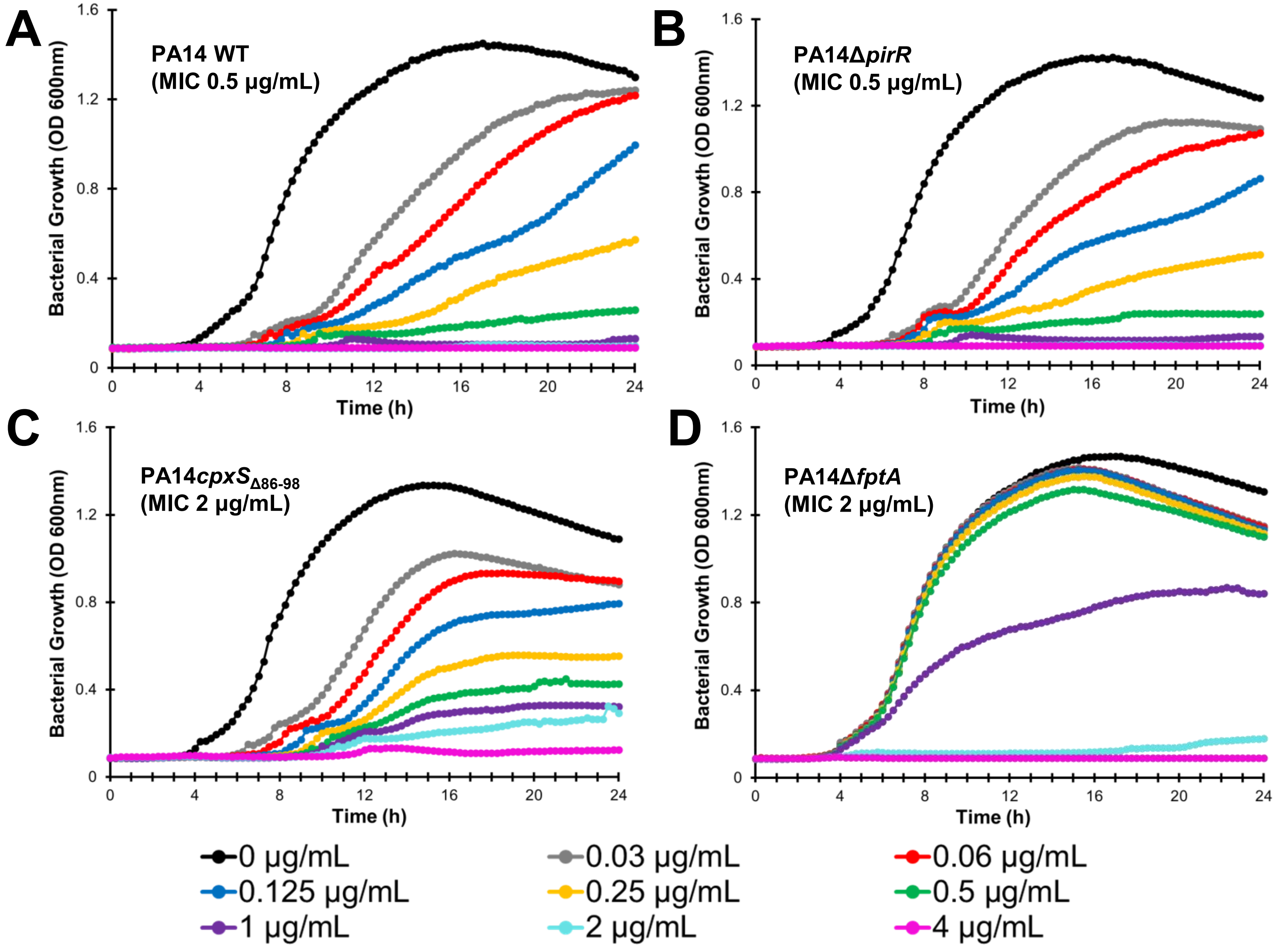

Figure S5. Inactivation of *fptA* abolishes bacteriostatic effects of FDC at subinhibitory concentrations. (A-D) Bacterial growth (O.D. 600 nm) measured every 30 min for 24 h in iron-depleted Mueller-Hinton broth with increasing concentrations of FDC (0.03 – 32 µg/mL) for PA14 WT **(A)**, PA14Δ*pirR* **(B)**, PA14*cpxS*$_{\Delta 86\text{-}98}$ **(C)**, and PA14Δ*fptA* **(D)**.

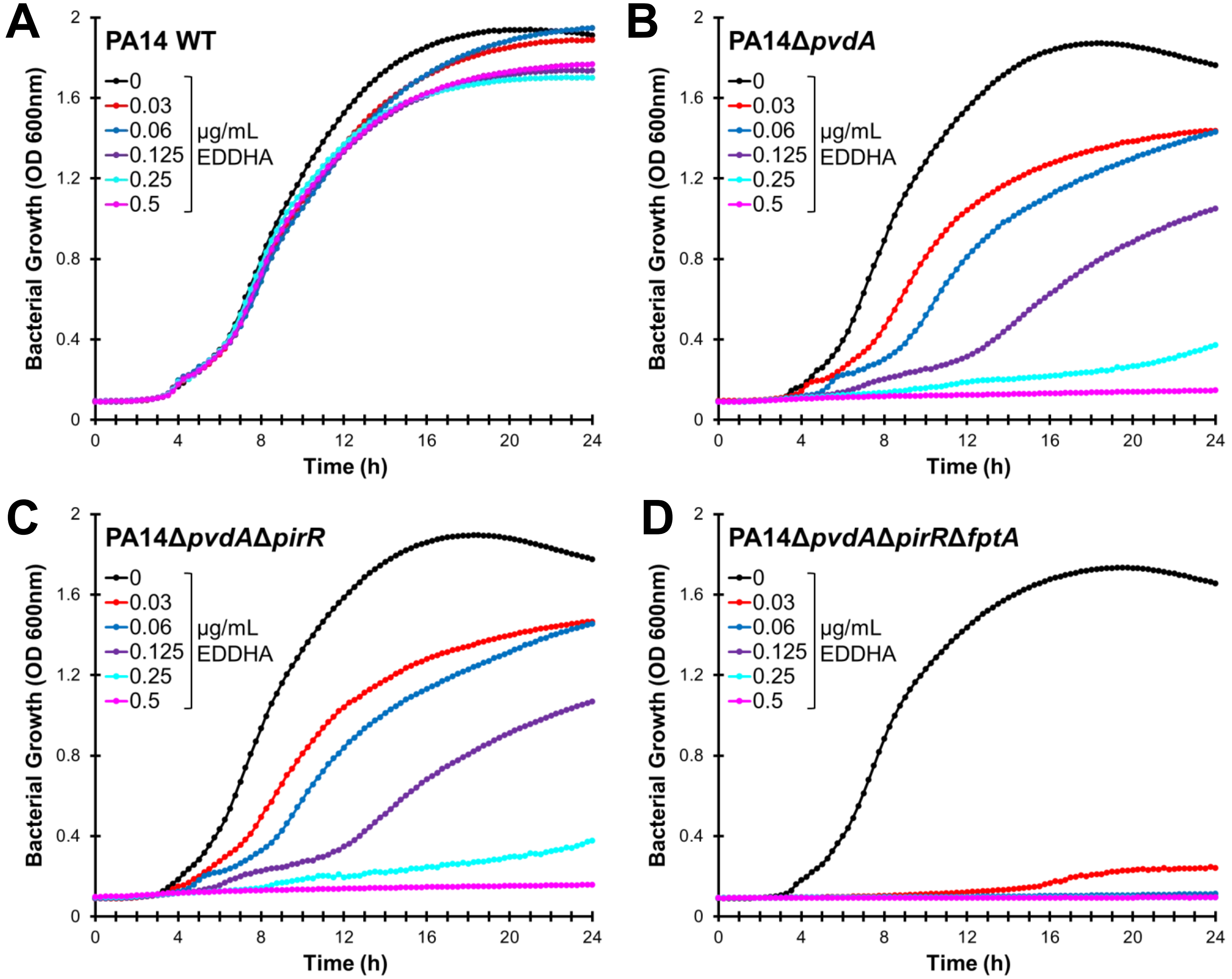

Figure S6. Inactivation of *fptA* confers a fitness cost in a pyoverdine biosynthetic mutant under severe iron starvation. (A-D) Bacterial growth (O.D. 600 nm) measured every 30 min for 24 h in Mueller-Hinton broth (not iron-depleted) with increasing concentrations of ethylenediamine-N,N′-bis(2-hydroxyphenylacetic acid) (EDDHA) (0.03 – 0.5 µg/mL) for PA14 WT **(A)**, PA14Δ*pvdA* **(B)**, PA14Δ*pvdA*Δ*pirR* **(C)**, and PA14 Δ*pvdA*Δ*pirR*Δ*fptA* **(D)**.

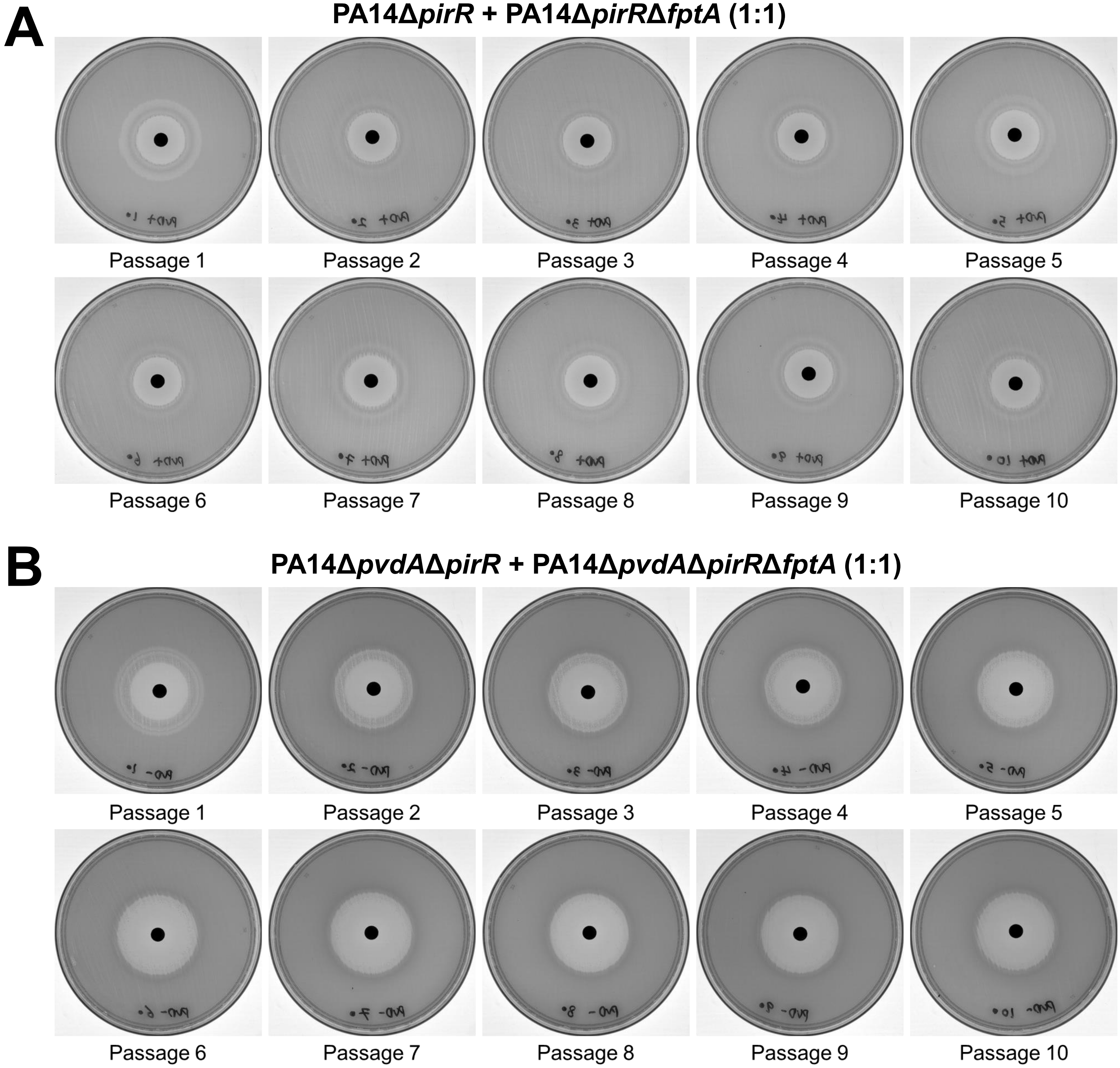

Figure S7. Mixed population of *fptA*$^{+}$ and *fptA*$^{-}$ mutants is gradually resensitized to FDC in the absence of pyoverdine production. (A, B) Images of FDC disk diffusion testing plates for co-cultures of PA14Δ*pirR* and PA14Δ*pirR*Δ*fptA* **(A)** or PA14Δ*pvdA*Δ*pirR* and PA14Δ*pvdA*Δ*pirR*Δ*fptA* **(B)** passaged in iron-depleted Mueller-Hinton broth for 10 days.
